# Review: The Consumption of Ultra-Processed Foods and Non-communicable Diseases in Latin America

**DOI:** 10.3389/fnut.2021.622714

**Published:** 2021-03-24

**Authors:** Rodrigo A. Matos, Michelle Adams, Joan Sabaté

**Affiliations:** ^1^EP Ingeniería de Industrias Alimentarias, Facultad de Ingeniería y Arquitectura, Universidad Peruana Unión, Lima, Peru; ^2^Center for Nutrition, Healthy Lifestyle, and Disease Prevention, School of Public Health, Loma Linda University, Loma Linda, CA, United States

**Keywords:** ultra-processed foods, chronic disease, metabolic disease, cardiovascular disease, diabetes, Latin America

## Abstract

The objective of this article is to assess current trends in Latin America with respect to the consumption of ultra-processed foods and non-communicable diseases. This review addresses the rapid growth of the ultra-processed foods market in Latin America which, along with other social and environmental factors, has been shown to be highly influential in the prevalence of non-communicable diseases such as obesity, type 2 diabetes, hypertension and cardiovascular disease, cancer, and all-cause mortality. Ultra-processed foods represent a health concern for a number of reasons. They are generally calorically dense and high in sodium, sugar, and saturated and *trans* fats, and low in fiber and protein. Additionally, they may contain additives and neoformed compounds that affect health in ways that have not been adequately researched. Furthermore, the packaging of ultra-processed foods may contain hormone disruptors whose effects on humans are not entirely clear. Associations between ultra-processed foods and cardio-metabolic dysfunction, as well as several plausible mechanisms, will be evaluated.

## Introduction

Ultra-processed foods (UPF) are food products that have been manufactured through the use of multiple industrial techniques (1). These techniques can include hydrogenation, extrusion, pre-frying and/or the addition of colorants, emulsifiers, and preservatives. Employing ultra-processing techniques allows manufacturers to create products that are hyper-palatable, cheap to produce, easy to market, and able to sit on store shelves or remain in the kitchen cabinet for years without spoiling.

While these foods are not new, their consumption is becoming increasingly widespread in Latin America. At the same time, the prevalence of excess adiposity and the cardio-metabolic sequelae commonly associated with it have also risen (2–4). Latin Americans are becoming increasingly westernized in their food preferences, and the health consequences of this cultural shift are overwhelmingly harmful.

Evidence from cross-sectional and prospective studies points to a strong association between UPF consumption and overweight/obesity (5–7), hypertension (8), cardiovascular disease (CVD) (9), type-2 diabetes (10), cancer (11), and all-cause mortality risk (12–14). UPF deliver a poor nutrient profile (15) and their additives disrupt gut function (16–18). The packaging, while attractive, may be adding additional health risks (19, 20). To date, only one randomized controlled trial assessing the impact of UPF on cardio-metabolic health has been published, and the findings suggest that UPF consumption can lead to passive overeating and subsequent weight gain (21).

The objective of this article is to assess current trends in Latin America with respect to UPF consumption and cardio-metabolic diseases, highlighting the importance of this unfavorable connection as well as possible mechanisms.

## An Overview of Ultra-Processed Foods

Multiple classification systems have been developed for the purpose of distinguishing the level of processing a food has received. For research purposes, the most commonly utilized system (22) is the NOVA food classification system (23). This system places foods into one of four categories: unprocessed/minimally processed, processed culinary ingredients, processed foods, and ultra-processed foods (Table 1).

**Table 1:** NOVA food classification system according to its level of processing.

**Food classification**	**Food example**
Unmodified or minimally processed foods	Fresh/frozen fruits and vegetables, fresh meat, fresh milk, grains, eggs, fresh fish, nuts, granola, rice, beans, tubers, whole grain flour, herbs and spices, etc.
Processed foods as processed culinary ingredients	Extracted vegetable oils, substances isolated or modified by various preservation methods, salt, sugar, oil, fat, flour, white rice, pasta, butter extracted from fresh milk, extracted honey, starches extracted from corn and other plants, etc.
Processed foods	Vegetables and legumes modified or preserved with additives, salty or sugary nuts and seeds, canned meats and fish, canned fruits, fresh whole grain breads, fresh cheese, etc.
Ultra-processed foods	Industrial formulas with multiple ingredients, including: soft drinks, energy drinks, fruit nectar drinks, alcoholic beverages, distilled beverages, beer, refined cereal, breads, ready-to-eat meals, instant cereals, cookies, candy, sugary drinks, margarine, mayonnaise, chips, instant soups, confectionery, jams, chocolate, ice cream, cake, energy bars, dairy drinks, yogurts, processed cheese, pizza, pasta dishes, instant sauces, processed meat products, meat analogs, infant formulas, weight loss products such as meal replacement shakes and powders, etc.

*Source: Adapted from Monteiro et al. (1)*.

Foods which carry the distinction of being ultra-processed share a few defining characteristics. From the point of view of the consumer, they are convenient, hyper-palatable, and cheap. From the manufacturer's point of view, these foods are economical to produce, easy to advertise, and have a long-shelf life, all of which favor profit. Nutritionally, high concentrations of sodium, sugar, hydrogenated oils, and additives are common to UPF. They tend to be energy dense and contain more saturated fat, *trans* fat, and free sugar along with lower levels of fiber, protein, sodium, and potassium when compared to minimally processed foods (24).

Critics of the NOVA system claim that its simplicity is inadequate to contribute to dietary guidelines, and that a dietary pattern which contains UPF may not necessarily be micronutrient-poor and hyper-palatable (25).

## Concerns Surrounding the Intake of Ultra-Processed Foods

Putting whole foods through any type of processing fundamentally alters their food matrix, typically in a detrimental manner. For one, UPF are generally dense in calories from sugar and saturated fat but poor in fiber and micronutrients, a combination which contributes unfavorably to a healthy diet pattern (15). They are also more likely to contain *trans* fats, which have been definitively classified as harmful for cardiovascular health (26).

The additives found in these foods also have questionable effects on health. Carrageenan and carboxymethylcellulose (CMC), thickening agents commonly employed in meat and dairy product formulations, have been shown to be associated with intestinal inflammation (18). It has been posited that the underlying mechanisms are likely to involve damage to the endothelial barrier, upregulation of pro-inflammatory cytokines, and interference with the immune response. Non-caloric artificial sweeteners, often used in the place of sugar in products that are advertised as being low in sugar or sugar-free, may also contribute to metabolic dysfunction by causing gut dysbiosis (16). Emulsifiers, a ubiquitous class of stabilizers commonly added to processed foods, have been shown to induce metabolic syndrome and colonic inflammation in mice (17).

The packaging of ultra-processed and processed foods adds an additional layer of concern. Synthetic compounds like bisphenol A (BPA) are omnipresent in food packaging, and BPA in particular has been shown to act as a xenohormone with the potential to impair reproductive function in men and increase cancer risk (19). While some manufacturers have responded to the public's concerns about BPA exposure by switching to bisphenol S (BPS), research indicates that this alternative may actually be more readily absorbed into the body (27). Phthalates are another class of synthetic chemicals commonly used in food packaging, and like BPA and BPS, they have the potential to act as xenohormones. Among individuals in the National Health and Nutrition Examination Survey (NHANES) 2013–2014 cohort, intake of UPF was correlated with increased exposure to phthalates (20).

Nevertheless, it is important to recognize that food processing has provided many benefits to society. Nutritious foods such as whole grain bread, canned beans, and frozen spinach are all examples of processed foods. Processes such as pasteurization decrease the risk of microbial contamination. Applying heat to food prior to consumption makes many foods easier to digest, and it has been theorized that cooking with fire contributed significantly to human evolution and advancement (28). Phytonutrients such as lycopene are significantly more bioavailable after heat-processing (29). The enrichment and fortification of processed foods provides micronutrients which certain vulnerable populations may have trouble consuming sufficient amounts of (30, 31). Additionally, many processed foods are designed to cater to specific sub-populations with chewing and swallowing difficulties, such as people living with dysphagia (32). Processing food is not inherently bad, but the displacement of minimally processed foods in favor of these foods warrants concern.

## Concurrent Trends in the Intake of Ultra-Processed Foods and Chronic Diseases in Latin America

**Figure 1:**
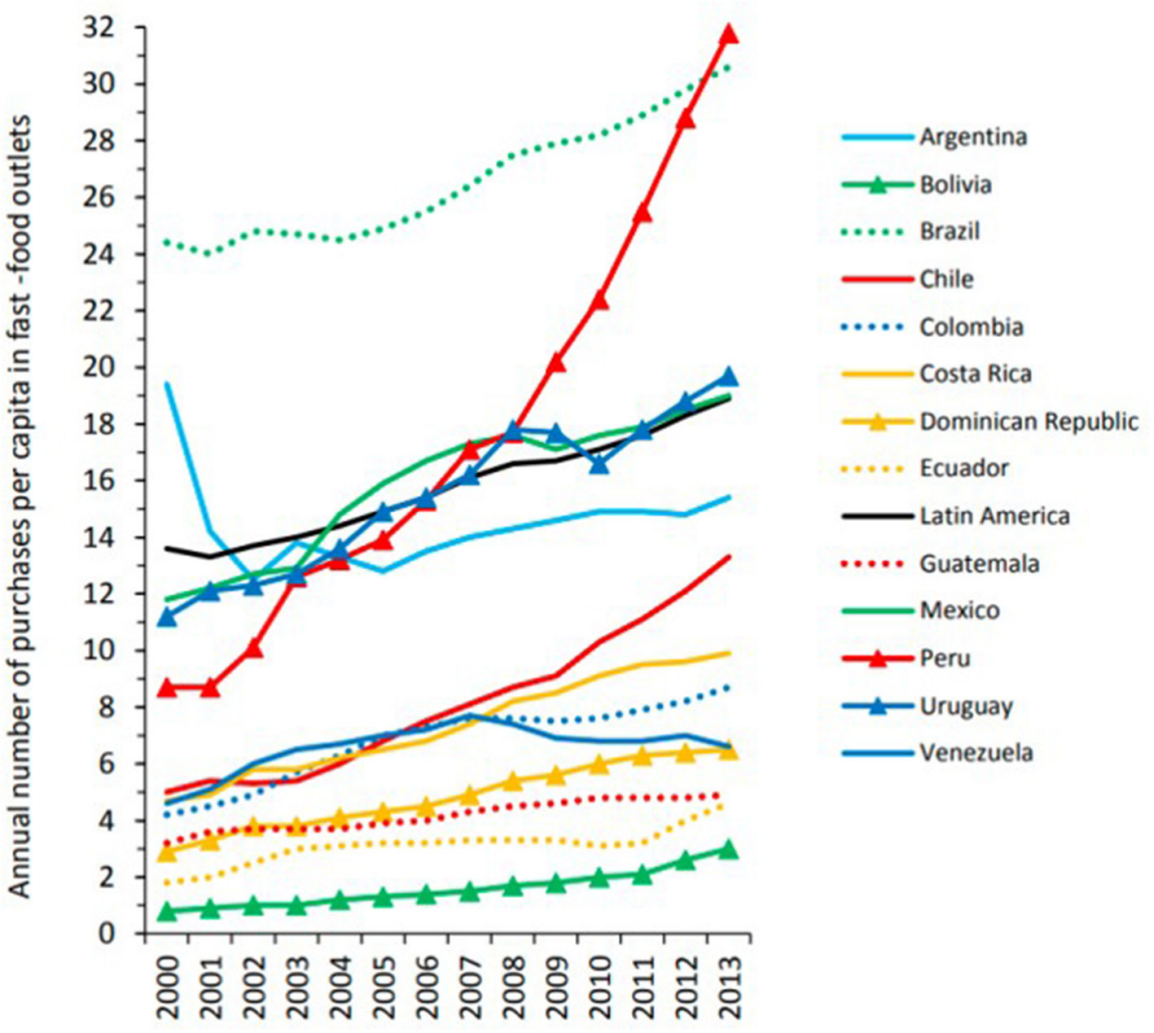
Annual number of purchases per capita in fast-food outlets in 13 Latin American countries, 2000–2013. Purchases refers to single, completed purchases (which may include more than one meal). Fast-food outlets are defined as establishments offering limited menus prepared quickly where customers order, pay, and pick up from a counter. Data are from the Euromonitor Passport Database (2014). Source (2).

The consumption of UPF is growing exponentially in Latin America countries (2). In a joint effort, the World Health Organization (WHO) and the Pan American Health Organization (PAHO) conducted an epidemiological study in 13 countries throughout Latin America with the goal of determining how the increasing prevalence of UPF in Latin American markets was affecting the chronic disease burden of these countries (2). The following countries were included in this study: Argentina, Bolivia, Brazil, Chile, Colombia, Costa Rica, Dominican Republic, Ecuador, Mexico, Peru, Uruguay, Venezuela, and Guatemala. Between 2000 and 2013, the retail sales of ultra-processed products, which encompasses both foods and beverages, from fast-food outlets increased in almost all 13 countries, with Argentina and Venezuela being the exception due to financial crises (Figure 1).

**Figure 2:**
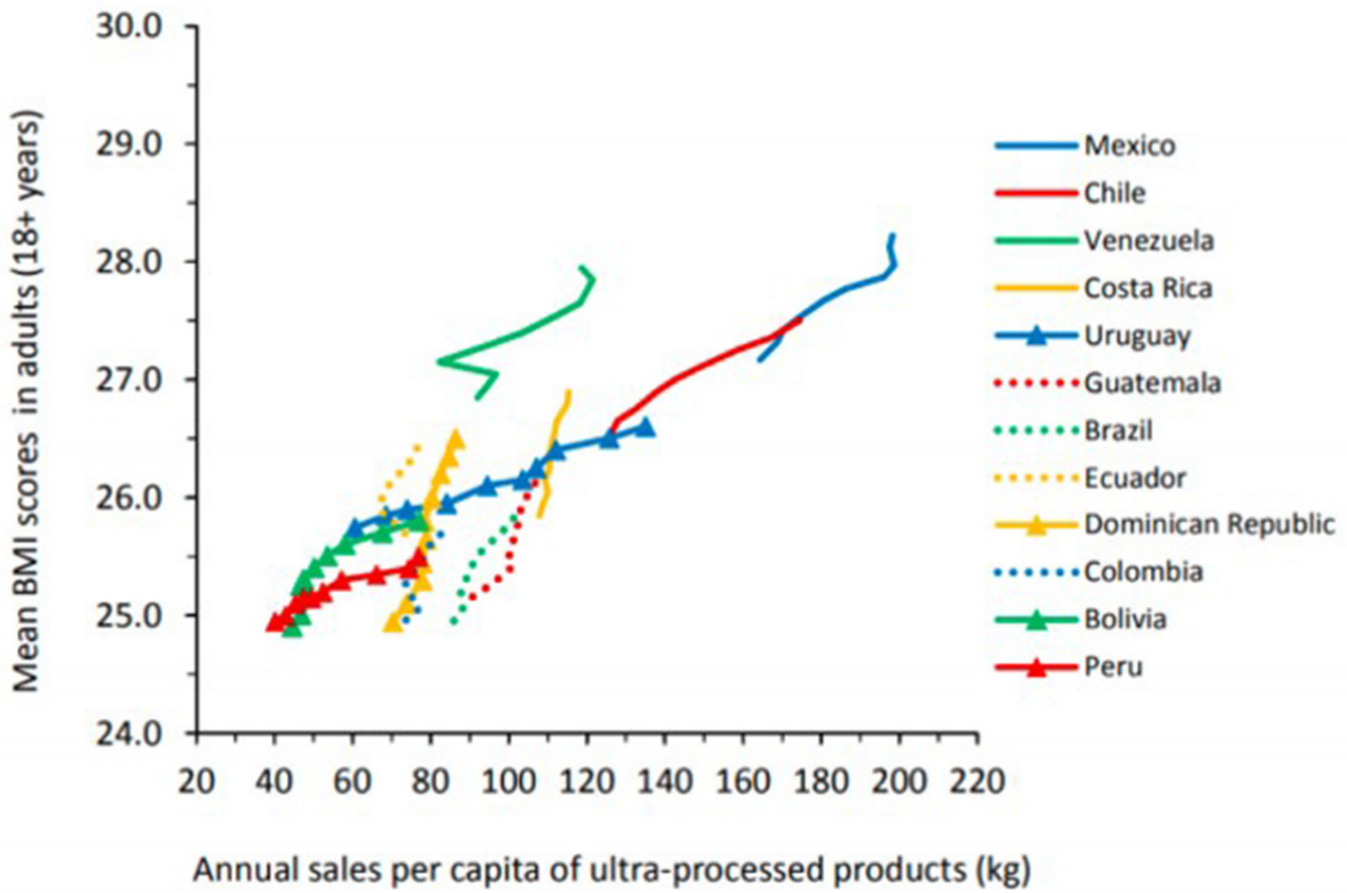
Annual sales per capita of ultra-processed food and drink products and mean body mass index (BMI) scores in 12 Latin American coutries, 2000-2009. Ultra-processed products here include carbonated soft drinks, sweet and savory snacks, breakfast cereals, confectionary (candy), ice cream, biscuits (cookies), fruit and vegetable juices, sports and energy drinks, ready-to-drink tea or coffee, spreads, sauces, and ready-meals. Source (2).

**Figure 3:**
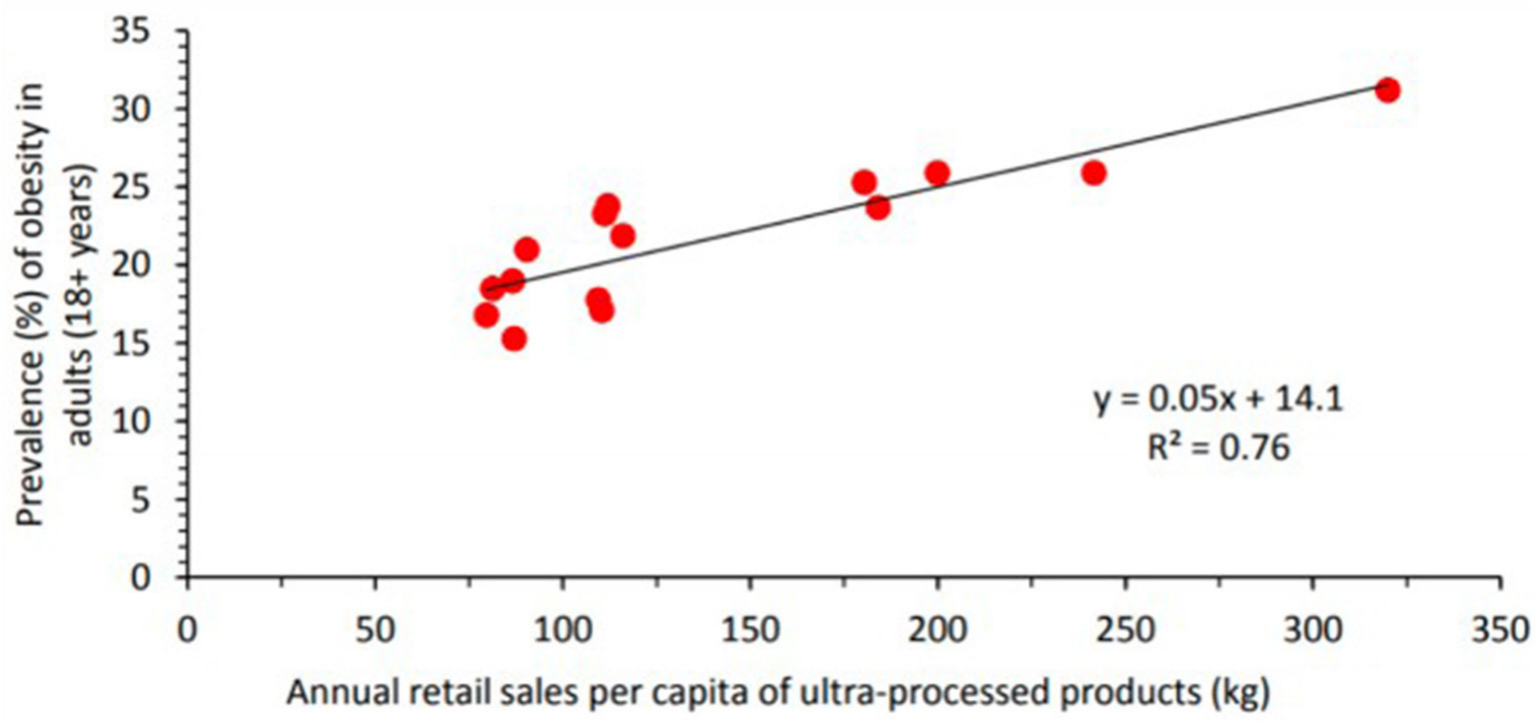
Annual retail sales per capita of ultra-processed food and drink products and prevalence of obesity (%) in adults in 14 countries (Bolivia, Brazil, Chile, Colombia, Costa Rica, Dominican Republic, Ecuador, Guatemala, Mexico, Peru, Uruguay, Venezuela, Canada, and the United States) in the Americas, 2013. Ultra-processed products here include carbonated soft drinks, sweet and savory snacks, breakfast cereals, confectionary (candy), ice cream, biscuits (cookies), fruit and vegetable juices, sports and energy drinks, ready-to-drink tea or coffee, spreads, sauces, and ready-meals. Source (2).

Overall, there is a positive association between annual retail sales of UPF and the prevalence of obesity in Latin America (2). Figures 2, 3 depict this correlation. In Brazil, the increasing intake of processed foods, sugar-sweetened beverages (SSBs), and refined carbohydrates has occurred in tandem with the rising prevalence of overweight and obesity in that country (3). These concurrent trends have been identified as major causes of death from cardio-metabolic diseases in Brazil. Between 2002 and 2009, intake of UPF in Brazilian households increased from 20.8% of total calories to 25.4% (33). As intake of UPF increased, intake of minimally processed foods also decreased.

As retail sales for fast foods have risen in Latin America, so has the prevalence of diabetes (34).

**Figure 4:**
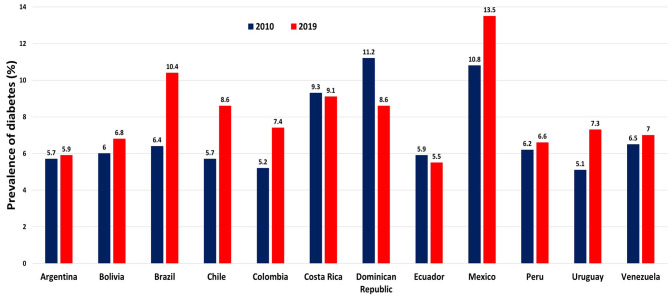
Prevalence of diabetes per country, 2010 and 2019. Data are from World Bank Database. International Diabetes Federation, Diabetes Atlas (2021). Source (34).

Within the span of the last decade, the prevalence of diabetes rose from 10.8 to 13.5% in Mexico, 6.4 to 10.4% in Brazil, and 5.7 to 8.6% in Chile (Figure 4). Argentina and Venezuela also experienced moderate (<1%) increases in diabetes prevalence. The only country to experience a notable decline in diabetes prevalence was the Dominican Republic, where there was a 2.6% reduction in diabetes prevalence. This ecological correlation between UPF sales per capita and diabetes prevalence invites discussion about a potential causal relationship between these two factors.

An assessment of diet quality conducted among children in the Bogotá School Children Cohort provides evidence that the nutrient profiles of UPF consumed in this region tend to be incongruent with good health (35). Specifically, these foods are lower in omega-3 polyunsaturated fatty acids, calcium, zinc, and vitamins A, C, E, and B12 when compared to unprocessed or minimally processed foods consumed in the same population. In Chile, UPF have been shown to contribute 58.6% (standard error 0.9%) of all added sugars consumed in the diet in a national sample (36). Soft drinks, fruit juices and flavored beverages, and cookies, cakes, and pies were the most commonly consumed high-sugar UPF among Chileans. Excessive consumption of added sugars has been correlated with increased cardiovascular disease risk (37).

This is concerning, particularly in the context of the latest data on food intake in Latin America which was collected as part of the Latin American Study of Nutrition and Health (ELANS) study (38). In this cross-sectional study, 24-h recall data from 9,218 adolescents and adults living in Argentina, Brazil, Chile, Colombia, Costa Rica, Ecuador, Peru, and Venezuela was assessed for intake of foods associated with non-communicable disease (NCD) risk. It was determined that <3.5% of respondents were meeting the WHO recommendations for intake of vegetables, whole grains, nuts, yogurt, and fish. A mere 7.2% of participants reported that they were consuming sufficient quantities of fruits and vegetables. If the trend in UPF intake continues, the number of Latin Americans who are eating a diet that is protective against NCDs will decline, and the prevalence of NCDs will continue to escalate.

While Venezuelans do not appear to be spending much on ultra-processed foods in the retail sector, it is worth noting that the typical Venezuelan diet still contains significant amounts of ultra-processed foods. Goodman and colleagues (39) collected data from a nationally representative sample of Venezuelans between 2014 and 2017 and found that traditional foods such as arepas and yucca remain staples in the Venezuelan diet and have not been replaced by more Western foods. In this country, Western fare like French fries and burgers are consumed once a month or less by three quarters of the population. Nevertheless, with a median consumption of one per day (interquartile range: IQR: 0.43–3 portions per day), the traditional foods that were most consumed in Venezuela—arepas and cheese—could still be classified as ultra-processed foods according to the NOVA classification system. Interestingly, there was a negative correlation between the prevalence of hypertension and type 2 diabetes and intake of arepas. This may be attributable to post-diagnostic changes in behavior.

## Social Determinants in the Sales Increase of Ultra-Processed Foods in Latin America

The following factors all contribute to the observed increase in the sale of ultra-processed foods in Latin-American countries: urbanization, foreign investment, and market deregulation. While the *volume* of sales of ultra-processed foods is higher in high-income countries like the United States, the *rate* of sales growth is higher in lower-income countries. It is evident that middle- and low-income countries are becoming increasingly attractive markets for the producers of ultra-processed drinks and foods.

The growing presence of UPF in grocery stores in Latin America places consumers in an environment that is challenging to navigate for several reasons (40). Firstly, relative to fresh foods, UPF tend to be sold at lower prices, an important factor for the millions of Latin Americans who are living in precarious financial situations. Secondly, the processed food labels often contain misleading health claims which may sway consumers to purchase a particular product with the aim of improving their health when in reality that product may be affecting their health negatively. And finally, food advertisers often aim their marketing tactics at children who are particularly susceptible to the effects of media (41). While television advertisements for ultra-processed foods and beverages abound on Latin American television, advertisements of minimally or unprocessed foods are difficult to come by (42).

Of note, the trends in intake of UPF vary country to country. As previously mentioned, economic crises have stymied the growth of the UPF market in Argentina and Venezuela (2). Meanwhile, daily per capita sales of SSBs is approximately 200 milliliters (ml) per capita per day in Colombia, while in Mexico that number is closer to 450 ml per capita per day (4). Per capita daily junk food sales is also twice as high in Mexico as it is for all of Latin America, while Chile is experiencing the fastest increase in sales of both SSB and junk food (4). While the overall sales volume varies widely between countries, the trend of reducing intake of traditional foods in favor of ultra-processed foods transcends nations.

## The Link Between the Intake of Ultra-Processed Foods and Chronic Diseases

The displacement of whole foods in the diet by UPF is becoming increasingly associated with all forms of disease risk.

### Overweight/Obesity

The association between the intake of UPF and the risk of being overweight or obese was assessed in numerous studies (Table 2), one of which is the NutriNet-Santé cohort study (5). After following 110,260 adults (mean age 43.1±14.6 years, 78.2% female) living in France for 10 years (2009–2019), researchers were able to conclude that intake of UPF is positively associated with risk of overweight (hazard ration (HR) for 10% absolute increase: 1.11, 95% confidence interval (CI): 1.08–1.14) and obesity (HR for 10% absolute increase: 1.09, 95% CI:1.05–1.13) after adjusting for energy intake, age, sex, education, physical activity, tobacco and alcohol use, marital status, and number of 24-h diet records. These findings are consistent with the findings of the Seguimiento Universidad de Navarra (SUN) Project, a Spanish prospective study wherein consumption of ultra-processed foods was also found to be associated with an increased risk of overweight and obesity (6). In another Spanish cohort, the Seniors Study on Nutrition and Cardiovascular Risk in Spain: Seniors-ENRICA-1 cohort (mean age 67.1±5.8 years, 44% female), it was found that consuming UPF increased participants' likelihood of having abdominal obesity, a risk factor for cardio-metabolic dysfunction (7).

**Table 2:** Studies assessing the association between UPF intake and excess adiposity.

**Authors**	**Year**	**Study Design**	**Population**	**Median follow up period (years)**	**Main Findings**
Beslay et al. (5)	2020	Prospective	NutriNet-Santé cohort: 110,260 French adults (age 43.1 ± 14.6 years, 78.2% ♀)	10	UPF intake is associated with risk of overweight (HR_10%_: : 1.11, 95% CI: 1.08–1.14) and obesity (HR_10%_: 1.09, 95% CI:1.05–1.13)
Sandoval-Insausti et al. (7)	2020	Prospective	Seniors Study on Nutrition and Cardiovascular Risk in Spain Seniors (ENRICA-1) cohort: 652 Spanish elderly adults (age 67.1 ± 5.8 years, 44% ♀)	6	UPF intake is associated with greater odds of developing abdominal obesity (OR: 1.62, 95% CI: 1.04–2.54)
Canhada et al. (43)	2020	Prospective	Brazilian Longitudinal Study of Adult Health (ELSA-Brasil) cohort: 11,827 Brazilian adults (age 51.3 ± 8.7 years, 55.0% ♀)	3.8	UPF intake is associated with being at an unhealthy weight (RR: 1.27, 95% CI: 1.07–1.50) as well as weight gain (RR:1.33, 95% CI: 1.12–1.58)
Nardocci et al. (44)	2020	Cross-sectional	Canadian Community Health Survey, cycle 2.2 respondents: 19,363 Canadian adults (age 45.99 ± 18.1 years, 49.1% ♀)	n/a	UPF intake is associated with greater odds of being obese (predicted OR: 1.32; 95% CI: 1.05–1.57)
Hall et al. (21)	2019	RCT	20 adults (age 31.2 ± 1.6 years, 50% ♀) randomly assigned to consume an ultra-processed diet or an unprocessed diet for a period of two weeks	n/a	Following an ultra-processed diet led to greater intake of calories (*p* < 0.0001), carbohydrates (*p* < 0.0001), and fat (*p* = 0.0004), as well as weight gain (0.9 ± 0.3 kg, *p* = 0.009)
Juul et al. (45)	2018	Cross-sectional	National Health and Nutrition Examination Survey (NHANES) respondents: a nationally representative sample of 15,977 American adults (age 41.9 ± 0.2 years, 50.6% ♀)	n/a	UPF intake is associated with significantly higher BMI and WC and increased odds of overweight (OR: 1.48, 95% CI: 1.25–1.76), obesity (OR: 1.53, 95% CI: 1.29–1.81), and abdominal obesity (OR: 1.62, 95% CI: 1.39–1.89)
Mendonça et al. (6)	2016	Prospective	Seguimiento Universidad de Navarra (SUN) Project cohort: 8,451 Spanish adults (age 37.6 ± 11.0 years, 64.9% ♀)	8.9	UPF intake is associated with increased risk of overweight/obesity (adjusted HR: 1.26; 95% CI: 1.10–1.45)
Louzada et al. (46)	2015	Cross-sectional	Brazilian Dietary Survey respondents: a nationally representative sample of 30,243 Brazilians; age in years, range (%): 10–19 (24.2%), 20–39 (41.3%), 40–59 (26.0%), and ≥60 (8.5%); 50.2% ♀	n/a	UPF intake is associated with greater odds of being obese (OR: 1.98, 95% CI: 1.26–3.12)

*RCT, randomized controlled trial; HR_10%_, hazard ratio per 10% increment; 95% CI, 95% confidence interval; OR, odds ratio; RR, relative risk; WC, waist circumference; Age reported as Mean ± SD unless indicated otherwise; ♀, female*.

Similar results were obtained from a longitudinal study conducted in Latin America by Canhada and colleagues (43). This cohort consisted of 11,827 participants living in Brazil (mean age 51.3±8.7 years 55.0% female). Mean follow up time was 3.8 years, and diet was assessed via food frequency questionnaire. After adjusting for a variety and social and lifestyle factors, a significant positive association between intake of UPF and being at an unhealthy weight [relative risk (RR): 1.27, 95% CI: 1.07–1.50] as well as risk of gaining weight (RR:1.33, 95% CI: 1.12–1.58) was detected in this cohort.

In a cross-sectional Brazilian study (*n* = 30,243), researchers found that adolescents and adults with the highest intake of UPF according to the Brazilian Dietary Survey had significantly greater odds of being obese [odds ratio (OR): 1.98, 95% CI: 1.26–3.12] (46). The validity of this association is further supported by a cross-sectional study of NHANES data collected from 15,977 American adults, wherein UPF intake was found to be associated with greater adiposity, especially for females (45). The same association has been reported in a Canadian study as well (44).

There has been only one randomized controlled trial assessing the effects of UPF on health to date (21). Twenty adults (mean age 31.2 ± 1.6 years, 50% female) were randomly assigned to consume an ultra-processed diet or an unprocessed diet for a period of 2 weeks. Following a 2-week wash out period, participants were assigned to the alternate diet for an additional 2 weeks. The meals provided to participants were matched for energy, macronutrients, fiber, sodium, and sugar content and participants were instructed to eat *ad libitum*. While following the ultra-processed diet, participants consumed more calories (*p* < 0.0001), carbohydrates (*p* < 0.0001), and fat (*p* = 0.0004) and experienced significant weight gain (0.9±0.3 kg, *p* = 0.009). While on the unprocessed diet, participants experienced significant weight loss (0.9±0.3 kg, *p* = 0.007).

### Hypertension and Cardiovascular Disease

In a prospective study of 14,790 Spanish adults with a mean follow-up period of 9.1 years, participants with the highest intake of UPF had the greatest risk of developing hypertension (HR: 1.21, 95% CI: 1.06–1.37) (8). The findings of this study, the SUN project, are especially robust considering that data collection commenced in 1999, bringing the total person-years for this cohort to 134,784. In a similar vein, results from the NutriNet-Santé French cohort also indicate that higher consumption of UPF is associated with increased risk for coronary heart disease (HR for absolute 10% increase: 1.13, 95% CI: 1.02–1.24), cardiovascular disease (HR for absolute 10% increase: 1.12, 95% CI: 1.05–1.20), and cerebrovascular diseases (HR for absolute 10% increase: 1.11, 95% CI: 1.01–1.21), even with adjustment for diet quality (9).

### Type-2 Diabetes Mellitus

Srour and colleagues of the NutriNet-Santé French prospective cohort also assessed the association between UPF intake and type-2 diabetes (T2D) incidence (10). Consumption of UPF was shown to positively correlate with incident type-2 diabetes, and this association persisted after adjustment for metabolic comorbidities and diet quality.

### Cancer

Consumption of UPF has also been tied to increased cancer incidence. Overall cancer risk and breast cancer risk were both found to increase in proportion to the amount of UPF in subjects' diets within the NutriNet-Santé French cohort (HR per 10% increase: 1.12, 95% CI: 1.06–1.18 and HR per 10% increase: 1.11, 95% CI: 1.02–1.22, respectively) (11).

### Mortality

UPF also appear to adversely impact mortality risk. Using data from a sub-set (*n* = 44,551, mean age 56.7±7.5 years, 73.1% female) of the NutriNet-Santé French cohort, researchers discovered that after adjusting for common socioeconomic and lifestyle confounders, higher intake of UPF was associated with an elevated risk of death from all causes (HR per 10% increase: 1.14, 95% CI: 1.04–1.27) after a median follow up time of 7.1 years (12). In the SUN Spanish cohort study, participants who reported the highest consumption of UPF had the highest risk of all-cause mortality (HR: 1.62, 95% CI: 1.13–2.33). This relationship was also dose-dependent (*p* for trend: 0.005) (13). The same association has been found among US adults (14).

## Potential Mechanisms Which may Explain the Link Between the Intake of Ultra-Processed Foods and Chronic Diseases

### Decreased Nutritional Quality: Energy Density, Sodium, Sugar, and Fiber

The high energy density of UPF may partially explain the association between UPF intake and excess adiposity. This is because the energy density of foods in one's diet is predictive of overall energy intake (47). Studies show that as people incorporate more energy dense foods into their diets, they typically do a poor job of adjusting their overall energy intake. This leads to what has been termed as “passive over-consumption (47).”

As intake of UPF foods increases, intake of saturated fat, free sugars, and sodium all rise while fiber, protein, and potassium intake fall (48). UPF also tend to be high in sodium relative to minimally processed foods. Independent of other dietary factors, high sodium intake has been identified as a contributor to death due to cardio-metabolic dysfunction (49, 50). The same holds true for SSBs, which are ultra-processed (49). SSB consumption may also increase one's risk of developing hypertension (51) and CVD (52). Furthermore, the consumption of added sugars is significantly associated with CVD mortality risk (37). It has been estimated that 89.7% of energy provided by added sugars in a typical American diet comes from UPF (53).

The low fiber content of UPF may also play a role in the link between UPF intake and cardio-metabolic disease. Based on data compiled in an umbrella review of 18 meta-analyses, Veronese and colleagues found that high fiber intake was protective against cardiovascular disease incidence and mortality, coronary artery disease, and cancers associated with the gastrointestinal system (54). Ultra-processing typically removes the protective fiber layer of grains.

### Contaminants and Neoformed Compounds

Acrylamide, acrolein, polycyclic aromatic hydrocarbons (PAH), and furan are some common compounds associated with the preparation of UPF. Acrylamide is an organic compound that is formed when starchy foods are cooked at high temperatures. Potato chips and breakfast cereals are two examples of foods that may contain high amounts of acrylamide (55). Acrylamide may have adverse effects on hormone regulation, signal propagation across nerve fibers, reproductive health, and cancer formation, however this evidence comes primarily from animal studies (55). Dietary exposure to acrylamide as assessed by hemoglobin biomarkers was shown to be significantly associated with death from all causes in the NHANES 2003–2006 cohort (*p* for trend =0.0124) (56).

Acrolein is an unsaturated aldehyde that is produced as a result of cooking fats at high temperatures. In the Louisville Healthy Heart Study, acrolein exposure measured via a urinary biomarker was associated with increased CVD risk (57). PAH are produced during dry, high-heat cooking. When solid fuels such as wood or coal are used to prepare food, PAH from the burning of those fuels can contaminate the food. According to data from the NHANES 2005–2014 cohort, PAH exposure positively correlates with diabetes prevalence (58). Similarly, Ranjbar and colleagues determined that dyslipidemia, hypertension, excess adiposity, and type-2 diabetes may all be related to PAH exposure (59). Furan is an organic compound that is also produced during high heat cooking as a consequence of thermal degradation. The European Food Safety Authority has stated publicly that while there are a plethora of uncertainties in the risk assessment of furan, this compound has been shown to induce oxidative stress in animal models and has the potential to be hepatotoxic (60).

### Alteration of the Food Matrix

Processing foods alters the food matrix and often involves the removal of protective food structures. For instance, refined grains and their products are very low in fiber due to the removal of the bran layer. In animal studies, fiber intake has been shown to be protective against obesity in the context of a high fat diet (61). Likewise, dietary polyphenols, which are also greatly reduced during ultra-processing, have been demonstrated to fortify the gut microbiome against inflammation associated with a high fat diet (62).

Ultra-processing also breaks down cellular structures and frees nutrients in food from the compartments in which they are normally contained, producing acellular nutrients (63). Destruction of the cell wall facilitates rapid digestion and may also spur bacterial overgrowth in the small intestine and alter gut microbiome composition (64). A high proliferation of gut bacteria can precipitate low grade inflammation in the host, which can affect not only local tissues but also the entire organism, as bacteria associated with inflammation and metabolic disease can translocate and enter the systemic circulation (65, 66). The composition of the gut microbiome is largely reflective of the host diet, and high intake of simple sugars favors the growth of microorganisms that can metabolize this substrate. As a consequence, microbial diversity may shift to favor leptin resistance, hyperphagia, and the development of chronic diseases (64, 67).

## Conclusions

The rising popularity of ultra-processed foods in Latin America is significantly associated with the prevalence of non-communicable diseases in this region of the world. This association is graded and reflects increasing urbanicity and interplay with foreign markets in the Latin American economy. While countries like Mexico and Chile appear to be consuming the most UPF per capita, these findings represent a major public health concern for all of Latin America. It would be prudent of policy makers to design measures that facilitate production, promotion, and access to healthy foods in order to reverse this trend. Promoting good health practices enhances quality of life not only at the individual level, but ultimately at the global level.

## Author Contributions

RM, MA, and JS all contributed to this manuscript through conceptualization, acquisition and interpretation of relevant data, and preparation of the final approved paper. RM and MA contributed equally to the writing of this manuscript. All authors contributed to the article and approved the submitted version.

## Conflict of Interest

The authors declare that the research was conducted in the absence of any commercial or financial relationships that could be construed as a potential conflict of interest.
